# Supplementary material for: Knowledge of health benefits, availability, accessibility and consumption of indigenous foods by urban adults in the Cape Metropole, South Africa
Source: PLoS One. 2025 Dec 2;20(12):e0333306. doi: 10.1371/journal.pone.0333306 (PMC12671764; doi:10.1371/journal.pone.0333306)
Supplement: Attachment — Submitted filename: PONE-D-24-41163_Rebuttal letter.docx [file pone.0333306.s002.docx]

**Tuesday, 19 August 2025**

**Rebuttal letter**

**The responses are presented below each point .**

1.Please ensure that your manuscript meets PLOS ONE's style requirements, including those for file naming. The PLOS ONE style templates can be found at

https://journals.plos.org/plosone/s/file?id=wjVg/PLOSOne_formatting_sample_main_body.pdf and

https://journals.plos.org/plosone/s/file?id=ba62/PLOSOne_formatting_sample_title_authors_affiliations.pdf

**Response: We have ensured that the manuscript is complaint.**

2. Please note that funding information should not appear in any section or other areas of your manuscript. We will only publish funding information present in the Funding Statement section of the online submission form. Please remove any funding-related text from the manuscript.

**Response: Funding details have been placed in appropriate sections.**

3. We note that you have indicated that there are restrictions to data sharing for this study. PLOS only allows data to be available upon request if there are legal or ethical restrictions on sharing data publicly. For more information on unacceptable data access restrictions, please see http://journals.plos.org/plosone/s/data-availability#loc-unacceptable-data-access-restrictions.

**Response: The University of Stellenbosch requires that the following is completed and approved by the Health Research Ethics Committee (HREC) before sharing data.**

**HREC MTA/DTA requirements, processes and term sheet:**

- [Material Transfer Agreement (MTA) and Data Transfer Agreement (DTA) **HREC requirements and processes**](http://www.sun.ac.za/english/faculty/healthsciences/rdsd/Documents/Ethics/MTA_DTA%20Guidance%20for%20researchers_V2_23082023.docx)
- [Data Transfer Agreeement (DTA): Guidance for Researchers](http://www.sun.ac.za/english/faculty/healthsciences/rdsd/Documents/Ethics/DTA%20Guidance%20for%20Researchers_V1_2082023.docx)
- [**HREC Termsheet**: Material/Data Transfer Agreement](http://www.sun.ac.za/english/faculty/healthsciences/rdsd/Documents/Ethics/2017%20MTA%20or%20DTA%20-%20HREC%20TERM%20SHEET-updated%2015-04-2021_new%20logo.docx)

**Data will be made available following the University policies upon request.**

Before we proceed with your manuscript, please address the following prompts:

a) If there are ethical or legal restrictions on sharing a de-identified data set, please explain them in detail (e.g., data contain potentially identifying or sensitive patient information, data are owned by a third-party organization, etc.) and who has imposed them (e.g., a Research Ethics Committee or Institutional Review Board, etc.). Please also provide contact information for a data access committee, ethics committee, or other institutional body to which data requests may be sent.

**Response: Data requests may be sent to the corresponding author who is the data owner and will ensure compliance with the University policies for access as noted above. In addition, please note that in terms of the Protection of Personal Information Act No. 4 of 2013 of Republic of South Africa, Signed into law: 19 November 2013, published in the Government Gazette: 26 November 2013, Commencement dates: Full enforcement began on 1 July 2021b) there are also restrictions providing names of individuals of the Institutions other than committee details.** **Here is the contact details of HREC.** [**http://www.sun.ac.za/english/faculty/healthsciences/rdsd/Pages/Health-Research-Ethics.aspx**](http://www.sun.ac.za/english/faculty/healthsciences/rdsd/Pages/Health-Research-Ethics.aspx)

b. If there are no restrictions, please upload the minimal anonymized data set necessary to replicate your study findings to a stable, public repository and provide us with the relevant URLs, DOIs, or accession numbers. For a list of recommended repositories, please see

https://journals.plos.org/plosone/s/recommended-repositories. You also have the option of uploading the data as Supporting Information files, but we would recommend depositing data directly to a data repository if possible.

**Response: Not applicable.**

4. We note you have included a table to which you do not refer in the text of your manuscript. Please ensure that you refer to Table 3 and 4 in your text; if accepted, production will need this reference to link the reader to the Table.

**Responses: The comment has been implemented. It is actually Tables 2 and 3, and these have been referred to in the text and it is highlighted.**

5. If the reviewer comments include a recommendation to cite specific previously published works, please review and evaluate these publications to determine whether they are relevant and should be cited. There is no requirement to cite these works unless the editor has indicated otherwise.

**Response: The manuscript has been reviewed to ensure all citations are relevant.**

**Reviewers' comments:**

**Comments to the Author**

1. Is the manuscript technically sound, and do the data support the conclusions?

The manuscript must describe a technically sound piece of scientific research with data that supports the conclusions. Experiments must have been conducted rigorously, with appropriate controls, replication, and sample sizes. The conclusions must be drawn appropriately based on the data presented.

Reviewer #1: Yes

Reviewer #2: Yes

2. Has the statistical analysis been performed appropriately and rigorously?

Reviewer #1: Yes

Reviewer #2: I Don't Know

3. Have the authors made all data underlying the findings in their manuscript fully available?

The PLOS Data policy requires authors to make all data underlying the findings described in their manuscript fully available without restriction, with rare exception (please refer to the Data Availability Statement in the manuscript PDF file). The data should be provided as part of the manuscript or its supporting information, or deposited to a public repository. For example, in addition to summary statistics, the data points behind means, medians and variance measures should be available. If there are restrictions on publicly sharing data—e.g. participant privacy or use of data from a third party—those must be specified.

Reviewer #1: Yes

Reviewer #2: Yes

4. Is the manuscript presented in an intelligible fashion and written in standard English?

PLOS ONE does not copyedit accepted manuscripts, so the language in submitted articles must be clear, correct, and unambiguous. Any typographical or grammatical errors should be corrected at revision, so please note any specific errors here.

Reviewer #1: Yes

Reviewer #2: Yes

**Review Comments to the Author**

Please use the space provided to explain your answers to the questions above. You may also include additional comments for the author, including concerns about dual publication, research ethics, or publication ethics. (Please upload your review as an attachment if it exceeds 20,000 characters)

**Response: All responses to questions are in the affirmative.**

**Reviewer #1:**

Title: Knowledge of Health Benefits, Availability, Accessibility, and Consumption of Indigenous Foods by Urban Adults in the Cape Metropole, South Africa

The manuscript is well written and sounds very interesting. However, the researchers may need to consider some of the following comments and suggestions to improve the paper.

**Response: Thank you for the commendation.**

Some aspects of the paper are unclear, such as the difference between formal and informal supermarkets among the surveyed supermarkets. I think the type of supermarket may influence the food found, either exotic or indigenous. This is because various food products have different storage and transportation requirements. As a result, most informal supermarkets, particularly those owned by individuals in communities, lack the proper infrastructure to store certain food products, particularly perishable and semi-perishable products, which may lead to a poor supply of such products, regardless of whether they are IFs.

**Response:**

**Thank you for the comment. See lines 185 and 186. All supermarkets and trading stores were considered formal whether in town or township or owned by big companies of individuals or families. Informal refers to those selling as described below. Our results should be interpreted in that context. We agree that storage facilities will differ in the food establishments. Also note that food establishments such as Spaza shops are semi-formal to formal since they are rqired to register with the municipality and for Tax purposes, irrespective of the size. We do recognise the resource variation between the small and big, however, in the context of indigenous foods, these are mainly accessed from small establishments and informal vendors.** **However, the quality of fresh produce was seen to be medium to high amongst supermarkets and grocery stores, with a high quality observed in IFs sold at food stalls.**

**In the South African context, food establishments like supermarkets, grocery stores, and food stalls vary widely in formality, clientele, and cultural significance. Here's a breakdown of how we describe them and how they were used in this study:**

**Supermarkets – Formal**

**Examples: Checkers, Pick n Pay, Woolworths, Spar, Food Lovers market**

**Formality: Highly formal**

**Characteristics:**

- **Structured layout with standardized branding**
- **Operate under strict health and safety regulations**
- **Offer wide product ranges including fresh produce, packaged goods, and household items**
- **Staffed by uniformed employees with formal customer service protocols**

**Clientele: Middle to upper-income groups, urban and suburban shoppers**

**Payment Methods: Card, cash, mobile payments, loyalty programs**

**Grocery Stores – Semi-Formal to Informal**

**Examples: Local corner shops ("spaza shops"), independent grocers, convenience stores**

**Formality: Varies by location and ownership**

**Characteristics:**

- **Smaller scale than supermarkets**
- **Often family-run or community-based**
- **May not follow strict branding or layout standards**
- **Stock tailored to local preferences (e.g., maize meal, canned fish, bulk rice)**

**Clientele: Broad range, often serving lower- to middle-income communities**

**Payment Methods: Mostly cash, some accept mobile payments or cards**

**Food Stalls –Mainly Informal but some Semi-formal**

**Examples: Street vendors, market stalls, road stall, township braais, "shisanyama" spots, car washes and food stalls, weekend markets**

**Formality: Informal**

**Characteristics:**

- **Operate in open-air settings or temporary structures**
- **Sell ready-to-eat meals like chicken dust, *shisanyama*, *vetkoek*, boerewors rolls, bunny chow, *kota***
- **Often run by individuals or families without formal business registration**
- **Hygiene standards vary, though many are trusted by locals**

**Clientele: All income levels, especially in townships and urban centers**

**Payment Methods: Predominantly cash, some use mobile payment apps like SnapScan or eWallet**

**Cultural Note: In South Africa, informal food establishments play a vital role in food security and entrepreneurship. While supermarkets dominate in urban areas, informal vendors are deeply woven into the social and economic fabric of townships and rural communities.**

Another critical aspect was the distinction between income levels and the type of food items purchased among the participants.

**With regard to purchasing power, the income of households and the proportion they spent on food is discussed from 535 to 540.**

**We did not compare income levels and indigenous foods buying patterns.**

In most cases, affordability plays a crucial role in influencing buying patterns among communities; hence, the buying power in a given community also plays a part in determining the type of shops or supermarkets you could find. This is because low-income earners are most likely to buy key food products necessary to sustain them throughout the month, instead of purchasing healthy food products. Therefore, understanding the buying patterns based on income levels could help us understand why specific households prefer certain food products over others, particularly indigenous products over exotic ones.

**Response**: **Thank you for your comment. As explained above, we did not investigate expenditure patterns in this study as the aim focussed on accessibility and availability. We agree that in future studies we could focus on household patterns on household income, spending on indigenous foods and consumption.**

Furthermore, the study does not clearly explain the nutritional benefits of the IFs compared to the exotic ones readily available or accessible in the area or supermarkets using literature. Without the link between the IFs and their alternative foods offering similar benefits, it may be hard to say the participants prefer certain products over others, particularly if such products are not even related or used in place of each other, such as eating apples instead of guava. I think the products need to be comparable or offer the same benefits for one to say they prefer one over the other.

**Response: Nutritional benefits of Ifs have been added in the introduction second paragraph. Note that this was a cross-sectional study with no intervention and only investigated knowledge on health benefits of indigenous foods, and there was no comparing with exotic foods.**

Also, a link between the available IFs and their role in addressing malnutrition and non-communicable diseases, including their health benefits, would help show the health risks that could arise from the communities' eating habits.

**Response: Thank you, these aspects of IFs importance and contribution to food and nutrition security and no-communicable diseases is covered in paragraph one of the introductions.**

Abstract

Lines 29-32 – the background highlights the role played by different indigenous foods in addressing several problems, including diseases. Still, the food products covered in the study could not be linked to the above issues in the discussion.

**Response: A statement has been added in lines 405 to 407 as “Understanding the role of indigenous foods by the population and linking them to the health benefits such as prevention of non-communicable diseases, may help promotion of their consumption”.**

Line 33-35 – the aim mentions knowledge on the preparation and cooking of indigenous foods, but the study doesn't show any evidence of such a section being covered anywhere throughout the results.

**Response: Thank you for the observation, knowledge on acquisition, preparation and cooking of indigenous foods was investigated, however, it is not reported in this manuscript along with other constructs. The aim has been emended.**

Line 47- 50. The study's conclusion sounds too broad based on what was covered in the survey. I think the conclusion needs to align with the aim of the study, which will indicate if the surveyed communities possess the correct knowledge around the benefits of indigenous foods and the access or availability thereof. Now it's not clear if the potential is not realised because they don't consume the food or because they don't know its benefits.

**Response: the conclusion has been amended as per statement below.**

**Conclusion: The study population demonstrated limited knowledge regarding the definition and health benefits of IFs. Within the Cape Metropole, IFs were perceived as largely unavailable and inaccessible across food establishments. Additionally, there was a common misconception equating IFs with exotic foods. Despite this confusion, a significant proportion of participants expressed a willingness to purchase IFs if they were more readily accessible.**

Introduction

Line 64 – 113 correct sampling of district "sampling od districts,"

**Response: now line 129, corrected.**

Sampling

Line 114 correct that would be used for the piloting to “that was used”

**Response: amended now line 131.**

Sample size

Line 152 – be consistent, write ten percent in numbers and symbols

**Response: amended line 168 to 170.**

DISCUSSION

What makes the IFs better than the exotic foods, given that urban communities already buy exotic commodities, which could offer the same benefits? It would be better to promote the IFs so that the population can plant their own instead of just promoting that they buy the other in place of the other, unless the indigenous foods are cheap but overlooked due to a lack of knowledge.

**Response: You raise a valid and important point. Indeed, the promotion of Indigenous Foods (IFs) should ideally extend beyond encouraging purchase to fostering local cultivation and deeper cultural engagement, especially if these foods are affordable and nutritionally beneficial but underutilized due to limited awareness.**

**Our research aligns with this perspective; however, it was observational in nature and did not involve any intervention strategies. The study aimed to explore existing perceptions, knowledge levels, and accessibility of IFs within urban communities, rather than to influence behavior or promote specific practices. While our findings highlight the potential for greater uptake of IFs, particularly if barriers such as availability and awareness are addressed, further research would be needed to assess the impact of cultivation initiatives or educational campaigns.**

Indigenous food availability

Since IFs play a significant role in reducing non-communicable diseases, were the assessed IFs part of the important ones or not? If not, then what does that mean about the population in the area, particularly as far as diseases are concerned?

**Response: We agree that the availability of IFs has important implications for public health, particularly in relation to non-communicable diseases. However, our study took a comprehensive approach by investigating all IFs available within the region, irrespective of their direct relevance to NCD prevention or management. This inclusive assessment allowed us to capture a broader picture of the community’s relationship with IFs, highlighting not only nutritional gaps but also cultural, economic, and accessibility factors.**

What foods were planted in home gardens? There were no signs of IFs, and some gardens had IFs.

**Response: see lines 486 to 489. The majority of households reported not having a home food garden, with only one-quarter indicating that they maintained one. Among those with home gardens, over half cultivated exclusively exotic produce, rather than Indigenous Foods. The 93 household participants who had home food gardens were asked to list the foods grown in their home food gardens. The detail is not reported in this manuscript shows the food items that were grown in home food gardens. Forty-four participants reported growing only exotic foods in their home food garden, while 29 participants reported growing a mixture of exotic and IFs.**

Limitations of the study

Maybe an explanation around the supermarkets used, if they were formal or informal supermarkets, such terminology would help one to have a clear picture of the establishments used for the study, or the difference thereof. This needs to be clarified under methodology.

**Response: All supermarkets and grocery stores (including Spaza shops) used were categorised as formal (registered) food establishments irrespective of geographic location. Informal food establishments have been defined above. Please also note that the geographic location and clientele of the shops does influence what they stork for selling. E.g. tripe would be found in certain supermarkets but not in others.**

The IFs used might have been influenced by access, since only starch was available, with no vegetables. This could speak to the supermarket's standards and storage conditions for certain food Items. Other IFs are highly perishable; hence, many food stalls or supermarkets shy away from purchasing them to avoid wastage.

**Response: see lines 575 to 583. We acknowledge this insightful comment. It is indeed true that access plays a significant role in shaping the availability of IFs, particularly in urban retail settings. As noted, many IFs are highly perishable, which can discourage supermarkets and food stalls from stocking them due to concerns about storage and wastage.**

**It is also important to highlight that IFs are predominantly produced by small-scale and subsistence farmers, who often face significant barriers in accessing formal markets. Historical regulations and procurement practices continue to favor commercial producers, effectively excluding smallholder farmers from mainstream supply chains. We hope that increased consumer demand and awareness will help shift these practices, encouraging more inclusive sourcing and improved availability of IFs across food establishments.**

Conclusion and recommendation

To overcome economic burden, people are bound to buy essential food products and never worry about their health benefits. Considering that the participants were at various income levels, was there any difference in their buying patterns, such as buying more health foods than their counterparts? This does not come up under discussion.

**Response: Thank you for raising this important point. While we recognize that economic constraints often drive households to prioritize affordability over nutritional value, our study did not include a comparative analysis of household buying patterns across different income levels. As such, we are unable to draw conclusions about whether participants with higher incomes were more likely to purchase health-promoting foods than their counterparts. This remains an area for potential future research, particularly in understanding how socioeconomic status influences dietary choices and access to Indigenous Foods.**

Maybe a comparison between the IFs and exotic foods in terms of which they buy or eat in place of what IFs will help us understand if buying such foods is influenced by knowledge or its purely accessibility. Also, comparing prices will help us understand why they went for expensive, exotic foods over cheap, healthy ones.

**Response: Thank you for this valuable observation. Price comparisons were conducted at food establishments and are presented in lines 299 to 302 of the study. However, we did not collect household-level data comparing the prices of IFs and exotic foods, nor did we explore the decision-making processes behind food purchases. As a result, we are unable to determine whether choices were driven primarily by knowledge, accessibility, or perceived value. We acknowledge that this is a critical area for further investigation and recommend that future studies consider incorporating household-level pricing perceptions and purchasing behavior to better understand the dynamics influencing food choices.**

There's no link between the available IFs and their role in addressing malnutrition and non-communicable diseases, including their health benefits. Such a discussion will help us understand the health risks of the population.

**Response: Please refer to lines 60 to 68 and paragraphs 2 and 3 of the introduction on IFs roles and health benefits.**

**Reviewer #2:**

Dear Author,

Thank you for submitting your work for consideration for publication in PLOS One. The manuscript is relevant and sound, however, corrections will be needed to improve its quality. Below are my comments and suggestions:

(1) Abstract, very sound

**Response: Thank for your commendation.**

(2) Introduction, it is sound, however, authors must introduce IFs to the reader, what are they, nutritional composition, phytochemicals, examples of IFs in South Africa, and their contribution to the livelihood of households. Furthermore, previous studies done on IFs in South Africa should be included to show the knowledge gap and novelty of the current study. The objective of the study must be clear on line 81.

**Response: Thank you for the constructive feedback. We appreciate the recommendation to provide a clearer introduction to IFs. Please see the revised second and third paragraphs in the introduction, which now introduces the nutritional composition, phytochemical properties, and examples of IFs commonly found in South Africa. These sections also discuss their contribution to household livelihoods and cite previous studies conducted in the South African context to highlight the existing knowledge gap. Additionally, the objective of the study has been clarified on line 81 to ensure greater transparency and focus.**

(3) Methods, line 84 is not clear since authors indicated that the aim is to develop a profile of the status of IFs amongst adults between 18 and 80. How are they going to develop the profile? The statement must be revised. Line 96, remove "of the Cape Metropole" since it is a repetition of the previous line. Line 112, change "od" to "of districts". Line 114, is it "would be" or "was"? The same comment is applicable for lines 129 and 133.

**Response: The statement has been amended, see line 112 and the tense has been corrected on the other lines.**

(4) The interpretation of the results is very sound.

**Response: Thank you for the commendation.**

(5) Discussion, under indigenous food availability, authors should expand the discussion and explain the reason(s) for butternut, maize meal, samp and beans, samp and white-fleshed sweet potato as the most widely available IFs. The same is applicable for green leafy vegetables not featuring with regards to availability in this study. Otherwise, the discussion is very sound with scientific tone.

**Response: Please see highlighted section in the discussion.**

(6) Limitation of the study and conclusion, they are very sound.

**Response: Thank you for your commendation. The section has been revised.**
