# Supplementary material for: Knowledge of health benefits, availability, accessibility and consumption of indigenous foods by urban adults in the Cape Metropole, South Africa
Source: PLoS One. 2025 Dec 2;20(12):e0333306. doi: 10.1371/journal.pone.0333306 (PMC12671764; doi:10.1371/journal.pone.0333306)
Supplement: Attachment — Submitted filename: PONE-D-24-41163_Rebuttal letter_09 September 2025.docx [file pone.0333306.s003.docx]

**Tuesday, 09 September 2025**

**Rebuttal letter**

**The responses are presented below each point.**

Thank you for submitting your manuscript to PLOS ONE. After careful consideration, we feel that it has merit but does not fully meet PLOS ONE’s publication criteria as it currently stands. Therefore, we invite you to submit a revised version of the manuscript that addresses the points raised during the review process.

**Response: Thank you for giving us an opportunity to improve on the manuscript.**

The authors have satisfactorily addressed the reviewer comments. The data presentation is adequate, but I am concerned it will be a dry read as there are no Figures to complement the Tables.

**Response: We agree and have added two figures under methods.**

Under study design

- include coordinates of the "Cape Metropole". Some kind of map would also be useful.

**Response: We have added Figure 1 showing the Map of Cape Metropole and the districts as well as the coordinates.**

**Additionally, Figure 2 is added with visualisation for the multi-stage sampling process.**

Under data analysis

- Delete "Quantitative data was captured into a Microsoft Excel spreadsheet and cleaned by the principal investigator."

**Response: The statement has been deleted.**

- Specify the name, and version of the statistical software used.

**Response: Statistical Package for the Social Sciences IBM SPSS Statistics Version 29 has been inserted.**

Compiled by: XG Mbhenyane on behalf of the authors.
